# Distinct Roles for the Anterior Cingulate and Dorsolateral Prefrontal Cortices During Conflict Between Abstract Rules

**DOI:** 10.1093/cercor/bhw350

**Published:** 2016-11-22

**Authors:** Erica A. Boschin, Merima M. Brkic, Jon S. Simons, Mark J. Buckley

**Affiliations:** 1 Department of Experimental Psychology, University of Oxford, Oxford OX1 3UD, UK; 2 Department of Psychology, University of Cambridge, Cambridge CB2 3EB, UK

**Keywords:** anterior cingulate cortex, conflict-monitoring, dorsolateral prefrontal cortex, neuropsychology, Wisconsin Card Sorting Test

## Abstract

Distinct patterns of activity within the anterior cingulate cortex (ACC) and dorsolateral prefrontal cortex (dlPFC) reported in neuroimaging studies during tasks involving conflict between competing responses have often been cited as evidence for their key contributions to conflict-monitoring and behavioral adaptation, respectively. However, supporting evidence from neuropsychological patients has been scarce and contradictory. We administered a well-studied analog of the Wisconsin Card Sorting Test, designed to elicit conflict between 2 abstract rules, to a cohort of 6 patients with damage to ACC or dlPFC. Patients who had sustained more significant damage to the ACC were not impaired either on a measure of “conflict cost” nor on measures of “conflict-induced behavioral adaptation.” In contrast, damage to dlPFC did not affect the conflict cost measure but abolished the patients’ ability to adapt their behavior following exposure to conflict, compared with controls. This pattern of results complements the findings from nonhuman primates with more circumscribed lesions to ACC or dlPFC on the same task and provides converging evidence that ACC is not necessary for performance when conflict is elicited between 2 abstract rules, whereas dlPFC plays a fundamental role in behavioral adaptation.

## Introduction

In the last decade, the conflict monitoring and cognitive control (CMCC) model developed by Botvinick and colleagues (Botvinick et al. 2001; Botvinick 2007; Carter and van Veen 2007) has become an influential framework for the interpretation of behavioral, imaging, and neurophysiological data obtained from tasks involving interference between relevant and irrelevant dimensions of stimulus-response associations (i.e. conflict tasks). In conflict tasks, subjects are required to respond to the relevant dimension of a stimulus (e.g. its visual properties, such as color), while ignoring an irrelevant dimension (e.g. spatial location). The overlap between the relevant and irrelevant dimensions is manipulated so as to produce either facilitation in the response (e.g. if the color and the spatial location both cue the same response), or interference (if the color and the spatial location cue different responses), creating congruent/low-conflict, and incongruent/high-conflict trials, respectively. High-conflict (H) trials usually result in performance costs (i.e. conflict-effects or conflict-costs), with slower response times (RTs) and a higher error rate compared with low-conflict trials (L) (Simon and Small 1969; Hedge and Marsh 1975; Simon et al. 1981; Kornblum et al. 1990; Simon 1990; Lu and Proctor 1995).

According to the CMCC model, the presence of conflict is detected by conflict-monitoring units, which then, in turn, bias cognitive control units that allocate the resources necessary to implement conflict resolution. Once these resources have been engaged (i.e. on trials that directly follow H trials), the effects of conflict on performance are reduced, resulting in faster speed of response and higher accuracy, a phenomenon known as sequential-effects or conflict-induced behavioral adaptation. Imaging studies have often implicated the anterior cingulate cortex (ACC) and the dorsolateral prefrontal cortex (dlPFC) in the detection of conflict (Barch et al. 2000; MacDonald 2000; Braver et al. 2001; Peterson et al. 2002; Durston et al. 2003; Fan 2003; Hazeltine 2003; Weissman et al. 2003; Egner and Hirsch 2005; van Veen and Carter 2005; Chen et al. 2006; Sohn et al. 2007; Kim et al. 2011, 2012), and the implementation of cognitive control to aid behavioral adaptation (Durston et al. 2003; Egner and Hirsch 2005; Kerns 2006; Kim et al. 2012), respectively. As such, they are considered critical components of the model.

Kerns et al. (2004) and Kerns (2006) provide some of the most convincing evidence in support of the CMCC model, as the patterns of activation they describe very closely resemble the ones that would be predicted by the model, and they do so across 2 different tasks. In 2 separate studies, Kerns et al. observed conflict-related activation in the ACC during the Simon and Stroop tasks. ACC activation was higher on H trials than on L trials. High ACC activity also predicted good adaptation on subsequent trials. Furthermore, ACC activity was lower on H trials directly preceded by another high-conflict trial (HH) compared with H trials directly preceded by a low-conflict trial (LH), indicating that ACC is more active when conflict is first detected and less active when a higher level of cognitive control has already been engaged, such as in the case of HH trials. dlPFC, however, has been shown to be more active during adaptation trials characterized by faster responses on HH trials compared with LH trials (Egner and Hirsch 2005; Kerns 2006), indicating that this region is specifically active when cognitive control is proactively engaged in order to reduce the effects of conflict on performance. Furthermore, dlPFC activation was found to significantly correlate with ACC activation on the previous trial (Kerns 2006).

The imaging literature therefore provides correlational evidence supporting the CMCC model. However, while imaging studies may suggest whether a region is involved in a particular process, they cannot determine whether that region is necessary to support that process, and, as we outline below, evidence from neuropsychological studies have to-date failed to provide strong support for the CMCC model. Some studies involving patients with damage to ACC do indicate impairments in conflict tasks compared with controls (Cohen et al. 1999; Swick and Turken 2002; di Pellegrino et al. 2007; Sheth et al. 2012), whereas other studies report the opposite pattern of results, with no impairment in patients compared with controls (Vendrell et al. 1995; Stuss et al. 2001; Swick and Jovanovic 2002; Fellows and Farah 2005). Sheth et al. (2012) investigated the effect of dorsal ACC cingulotomy on a multiple interference task, and found that while cingulotomy did not affect performance during H trials, it did affect the patients’ ability to adapt on subsequent trials. Fellows and Farah (2005) investigated the effects of lesions to dorsal ACC in 4 patients carrying out the Stroop task, and found no impairments in response speed or accuracy. Patients were overall slower at the task compared with controls, but there were no significant differences in performance on H trials. Therefore, the evidence from neuropsychology regarding a crucial role of ACC in conflict monitoring and/or a modulatory influence on dlPFC to drive adaptation is markedly inconclusive.

**Figure 1.**
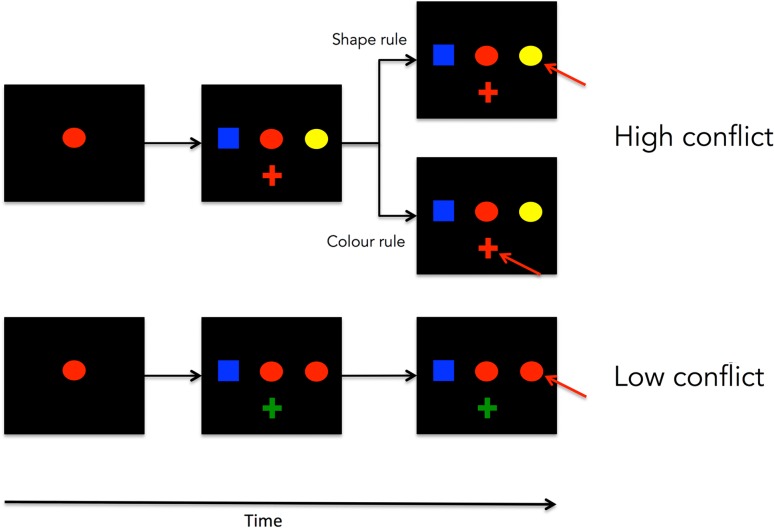
WCST conflict analog. An example of a typical trial in the WCST analog in the high-conflict condition (top) or the low-conflict condition (bottom). The correct choice is indicated by a red arrow.

Most of the aforementioned neuroimaging and neuropsychological studies used tasks whereby conflict is elicited by creating competition between a task-specific response (e.g. naming the color, in the case of the Stroop task) and a habitual/overlearned type of response that is often relevant and positively reinforced in contexts other than the task itself, therefore leading to a task-irrelevant predisposition for its selection (e.g. reading the word). However, conflict can also be elicited between 2 task-specific responses, such as in the case of the Wisconsin Card Sorting Test (WCST; Grant and Berg 1948). The WCST is a test that has been extensively used to assess the effects of prefrontal damage on cognition in patients (Milner 1963; Stuss et al. 1982) and has been shown to robustly recruit both the ACC and the dlFPC in imaging studies (Monchi et al. 2001; Buchsbaum et al. 2005; Lie et al. 2006; Specht et al. 2009). To investigate the role of the macaque ACC and dlPFC in conflict-monitoring and behavioral adaptation, Mansouri and colleagues (Mansouri et al. 2007, 2009, 2014, 2015; Kuwabara et al. 2014) have used an analog of the WCST for the monkey in which one of 2 uncued matching rules (“match by color” or “match by shape”) is reinforced at any one time (the reinforced rule changing without notice periodically) and the level of conflict between the 2 rules can be manipulated and randomly determined from trial-to-trial (see Fig. 1 and Materials and Methods for a more detailed description). Contrary to the CMCC's predictions, Mansouri and colleagues found that the behavior of animals with lesions to the ACC was unchanged both in terms of a classic conflict-cost effect (i.e. a faster RT observed on L than on H trials) and also in terms of conflict-induced behavioral adaptation (i.e. a faster RT on HH trials than LH trials). Animals with lesions to the dlPFC, however, while unchanged in the conflict-cost measure were impaired in the measure of conflict-induced behavioral adaptation with the adaptation effect usually observed on HH trials being abolished by the lesion. This is consistent with the idea that the dlPFC plays a crucial role in adaptation, but inconsistent with a fundamental and general role for ACC in all instances of conflict-monitoring and conflict-induced adaptation as proposed by CMCC theory.

In this study, we sought to investigate the role of ACC and dlPFC in conflict-monitoring and adaptation in humans by analyzing the performance of neuropsychological patients on the same WCST analog used by Mansouri et al. (2007) in their nonhuman primate study. By using the same experimental paradigm (both in terms of task and of effector systems) that has been used to investigate the effects of lesions to the analog areas of the monkey brain, we can avoid some of the confounds caused by the use of different methodologies in human (e.g. neuroimaging) versus nonhuman (i.e. single-cell recordings) primate studies, which are suggested to be partly responsible for the discrepancy between human neuroimaging literature and findings from animal work (Cole et al. 2010; Schall and Emeric 2010). Furthermore, a direct comparison with Mansouri and colleagues’ study by using the same task facilitates investigation of functional similarities or differences between the human and the monkey brain. Also, importantly, the conflict on H trials of the WCST analog is elicited between 2 task-specific rules (“match by color” and “match by shape”) that are equally reinforced and that would not be expected to elicit a strong task-irrelevant predisposition to select one over the other, as is the case in other conflict tasks where conflict is elicited between a task-specific response and a task-irrelevant habitual response. Using this task therefore allows us to investigate whether ACC is crucial for optimal performance in situations where conflict is generated between 2 task-specific responses, rather than when competition is between a task-irrelevant habit and a task-specific response.

If our hypothesis is correct that human dlPFC, but not ACC, is crucial for supporting conflict-induced behavioral adaptation only, paralleling what has been shown to be the case in monkeys (Mansouri et al. 2007), then we should expect to observe a significantly reduced adaptation effect in dlPFC patients than in controls, whereas we should not expect to see any such change in adaptation in ACC patients. In addition, we should expect to see no change in conflict-cost in either patient group relative to controls. In contrast, if, consistent with CMCC theory, ACC is indeed a crucial region for supporting general purpose conflict-monitoring, then we should instead expect to observe a larger conflict-cost effect in ACC patients than in controls. Accordingly, if ACC drives adaptation on subsequent trials we would also expect to see a smaller adaptation effect in ACC patients than in controls.

## Materials and Methods

### Participants

Eighteen participants (15 male, mean age 62.95 years) took part in the study. Of these, 6 participants were included the lesion groups, who were recruited through the Cambridge Cognitive Neuroscience Research Panel and through the volunteer panel of the Psychology Department at the University of Birmingham. Three patients with damage to the ACC were assigned to the ACC lesion group and 3 patients with damage to the dlPFC were assigned to the dlPFC lesion group. The average period between the onset of the damage and testing was 131 months (range: 53–168 months). For each patient, 2 control participants, matched on age, gender, and National Adult Reading Test (NART) scores, were recruited through the volunteer database at the MRC Cognition and Brain Sciences Unit in Cambridge, as well as from advertisement in the Oxford area. Control participants were not taking any psychoactive medication and were free of current or past neurological or psychiatric conditions, as determined by their history. All subjects had normal or corrected-to-normal vision. All subjects were native English speakers and provided written consent prior to their participation in the study in a manner approved by the Cambridge Psychology Research Ethics Committee and South Birmingham NRES committee.

### Lesions

Lesions were traced from MR or CT images and mapped onto the standard Montreal Neurological Institute (MNI) brain using MRIcro software (Rorden et al. 2007). A detailed summary of the extent and location of the lesions is provided in the Supplementary Material (see Supplementary Tables S1, S2, and S3). The aetiologies included meningioma, aneurysm, and encephalitis. A summary of the participants’ background information, including etiology of the damage, is included in Table 1.

**Table 1:** Patients’ demographic information, including etiology, total lesion volume and percentage of the total lesion volume localized to the specific areas of interest

Patient ID	Age	Gender	Group	NART IQ	Area(s)	Volume (mm^3^)	Etiology
1	69	M	ACC	121.57	24 + 32R	6320	Meningioma
2	65	M	ACC	93.09	32L	4128	Meningioma
3	58	M	ACC	109.11	24 + 32	7315	Encephalitis
4	62	F	dlPFC	123.35	46R	5320	Aneurysm
5	66	M	dlPFC	117.12	46L	464	Aneurysm
6	54	M	dlPFC	120.68	46L	6760	Meningioma

Notes: L, left; R, right. Detailed information about the extent and anatomy of the lesions is provided in Supplementary Tables S1, S2, and S3.

**Figure 2.**
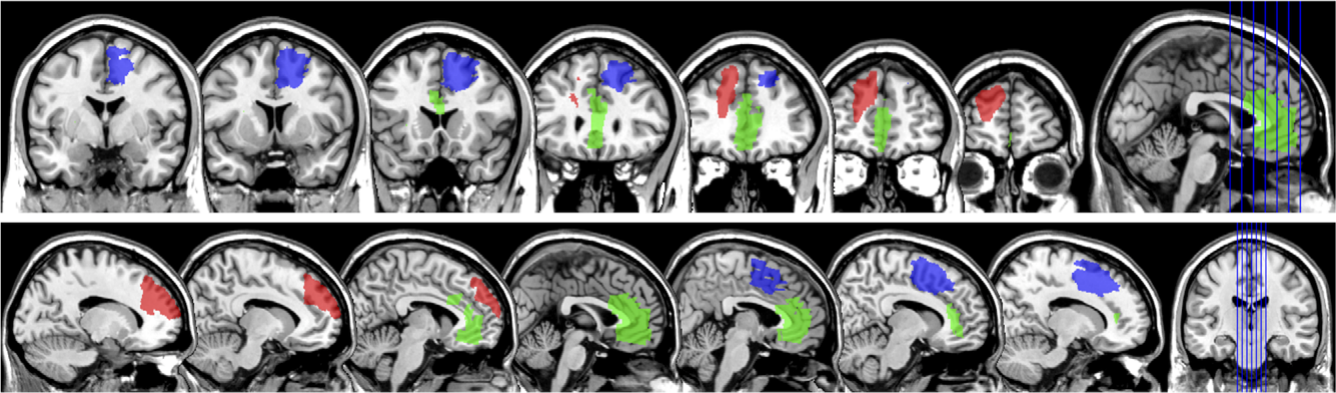
ACC lesions. Location and extent of ACC lesions in the 3 ACC patients included in this study, presented in a coronal (top, from posterior to anterior) and sagittal (bottom, from left to right hemisphere) view of a standard MNI brain. Each color denotes a different patient. Patient 1, blue; patient 2, red; patient 3, green.

**Figure 3.**
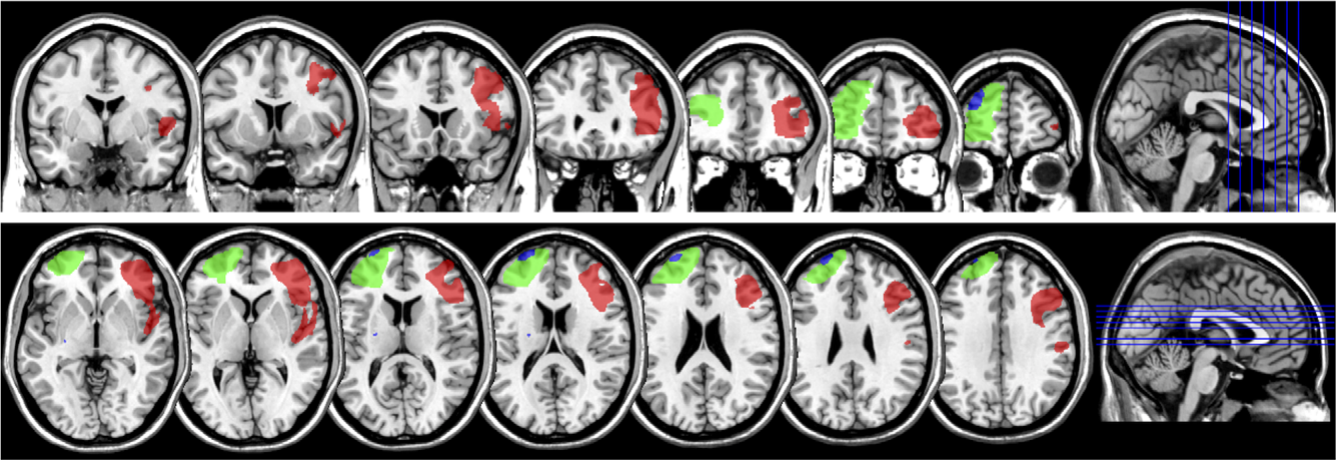
dlPFC lesions. Location and extent of dlPFC lesions in the 3 dlPFC patients included in this study in coronal (top, posterior to anterior) and horizontal (bottom, ventro-dorsal) views on a standard MNI brain. Each color denotes a different patient. Patient 4, red; patient 5, blue; patient 6, green.

ACC damage across participants was widespread and encompassed Brodmann areas 32 and 24. Patient 1 presented damage to dorsal portions of area 24, encroaching slightly onto 32 and pre-SMA, on the right hemisphere, patient 2 presented damage to dorsal and more anterior parts of area 32 on the left hemisphere, and patient 3 presented bilateral damage to more subgenual parts of ACC (see Fig. 2). There was no lesion overlap across ACC patients. dlPFC damage encompassed for the most part Brodmann area 46, and, to a lesser extent, area 9. Two patients (patients 5 and 6) presented damage to left dlPFC and patient 4 presented damage to right dlPFC (see Fig. 3). The area affected in patient 5 overlapped entirely with a portion of the larger lesion of patient 6.

### Apparatus, Stimuli, and Procedure

The task used in the study was a computerized version of WCST analog with trial-by-trial manipulations of conflict, originally developed by Mansouri et al. (2007). The task was programmed using Turbo Pascal (Borland), run in DOS on a desktop PC and presented on a 20.1″ color touchscreen (TFT LCD TS200H GNR), which was used to collect responses. Participants sat at a distance of 40 cm from the screen and were instructed to respond using the index finger of their dominant hand to touch the items on the screen.

The stimulus set consisted of all combinations of 6 possible shapes (triangle, circle, square, hexagon, ellipse, or cross), each 2.4° of visual angle in width and 2.4° in height, in 6 possible colors (red, green, blue, cyan, magenta, or yellow), for a total of 36 possible stimuli, and were presented against a black background. The sample item was always presented in the center of the screen, and the test items were presented 2.6° to the right, left, and bottom of the sample item (Fig. 1).

A typical trial was as follows. At the start of the trial, a random sample item was presented in the center of the screen. Participants were instructed to touch the sample item when they were ready to start the trial. Once the sample item was touched, 3 test items appeared on the screen. Participants were instructed to carry out a matching-to-sample task, where the rule for matching could be either “match by shape” (i.e. pick the test item that shared the same shape as the sample item) or “match by color” (i.e. pick the test item that shared the same color as the sample item). All items remained on the screen until a response was made or until 10 s had elapsed. Correct trials were identified by a high-pitch sound and the correct choice item remaining on the screen longer while the irrelevant items disappeared, indicating positive feedback. Incorrect trials were identified by a low-pitched sound accompanied by the presentation of a large, gray circle, indicating negative feedback. After 2 s from response, another sample item appeared on the screen, indicating the start of another trial.

Conflict levels were manipulated by changing the degree of feature overlap between the sample and test items. In low-conflict (L) trials, one of the test items was identical to the sample item (i.e. matched the sample item on both the relevant—e.g. color—and irrelevant—e.g. shape—dimension), while the other 2 test items shared neither shape nor color with the sample item. In high-conflict (H) trials, one of the test items matched the sample item only on the relevant dimension (e.g. color), while another matched the sample item only on the irrelevant dimension (e.g. shape). A third test item shared neither color nor shape with the sample item. H and L trials were presented in a randomized order throughout the session irrespective of the currently reinforced rule (examples of a H and a L trial are presented in Fig. 1).

Participants were informed that one rule would be “correct” for several trials and then the other would be “correct” for several trials, with the rules switching unpredictably during the task, such that they would have to periodically reassess which rule was currently relevant in order to perform the correct response. The rule switch occurred only once an accuracy criterion of 85% on the current rule had been reached over the preceding 20 trials. Participants carried out the task for 12–15 min, for a total of approximately 150 trials. Reaction times and errors were recorded for analysis.

## Results

All analyses were carried out on the speed of target selection (STS), which was computed by taking, on a trial-by-trial basis, the reciprocal of the RT data. We used this measure in order to minimize the potential impact of occasional high RT values (without the need to set any arbitrary criteria for removing potential outliers from the analysis) on the RT distribution, but, importantly, also to use the same unit of measurement previously used by Mansouri et al.’s (2007) macaque study, to which we wished to make direct comparisons. Therefore, it is useful to remember throughout that high STS values simply correspond to fast responses (i.e. higher speed), and low STS values simply correspond to slow responses (i.e. lower speed). STS analysis was carried out on correct trials only (and hence pairs of consecutive correct trials in the adaptation analyses). As the proportion of correct responses was very high, with the vast majority of errors restricted to switch trials (the average percentage of errors that were nonswitch trials was 3.16% for patients and 3.43% for controls), our analyses were carried out on STS only (and not accuracy, or number of switches), given the insufficient number of data points for error trials to carry out meaningful statistical analysis. We felt this was appropriate also given that Mansouri et al. (2007) found an effect of lesions on performance only for STS values, but not errors (see Mansouri et al. 2007, Supplementary Fig. S7).

In order to test the presence of conflict and adaptation effects, a 2 × 2 repeated-measures ANOVA was carried out on the STS from our control population, with Current Conflict (High and Low) and Previous Conflict (High and Low) as within-subject factors. As expected, there was a significant main effect of Current Conflict [*F*(1,11) = 38.45, *P* < 0.001], with L trials showing higher STS values (mean = 1.17, SD = 0.27) than H trials (mean = 1.07, SD = 0.27). This confirms the presence of a robust conflict effect across our population. The main effect of Previous Conflict did not reach significance [*F*(1,11) < 1]. Importantly, there was a significant interaction between Current Conflict and Previous Conflict [*F*(1,11) = 6.03, *P* = 0.032], confirming the presence of an adaptation effect, with the effect of conflict on the previous trial carrying over to the subsequent trial. Bonferroni-corrected paired-samples *t*-tests were carried out to investigate the significant interaction between Current and Previous Conflict. There was a significant difference between STS for HH and LH trials [*t*(11) = 2.53, *P* = 0.028], but no significant difference between HL and LL trials [*t*(11) < 1]. STS values for HH trials were larger (mean = 1.09, SD = 0.27) than for LH trials (mean = 1.05, SD = 0.28). This indicates that the same conflict-induced behavioral adaptation effect previously observed in macaques (Mansouri et al. 2007) is present in humans too when they perform the identical task, and it confirms that the adaptation effect is due to better adjustment (speed increase) on H trials following H trials (HH), rather than differences in response speed on L trials.

In order to investigate the effects of lesions on performance, the means of each patient group were directly compared with the means of their matched control group (each patient was matched with 2 controls) on our measures of conflict cost (i.e. STS of H vs. L) and conflict-induced behavioral adaptation (i.e. STS of HH vs. LH). Given the small sample sizes and non-normal distribution of the data, this was achieved via a series of nonparametric tests.

**Figure 4.**
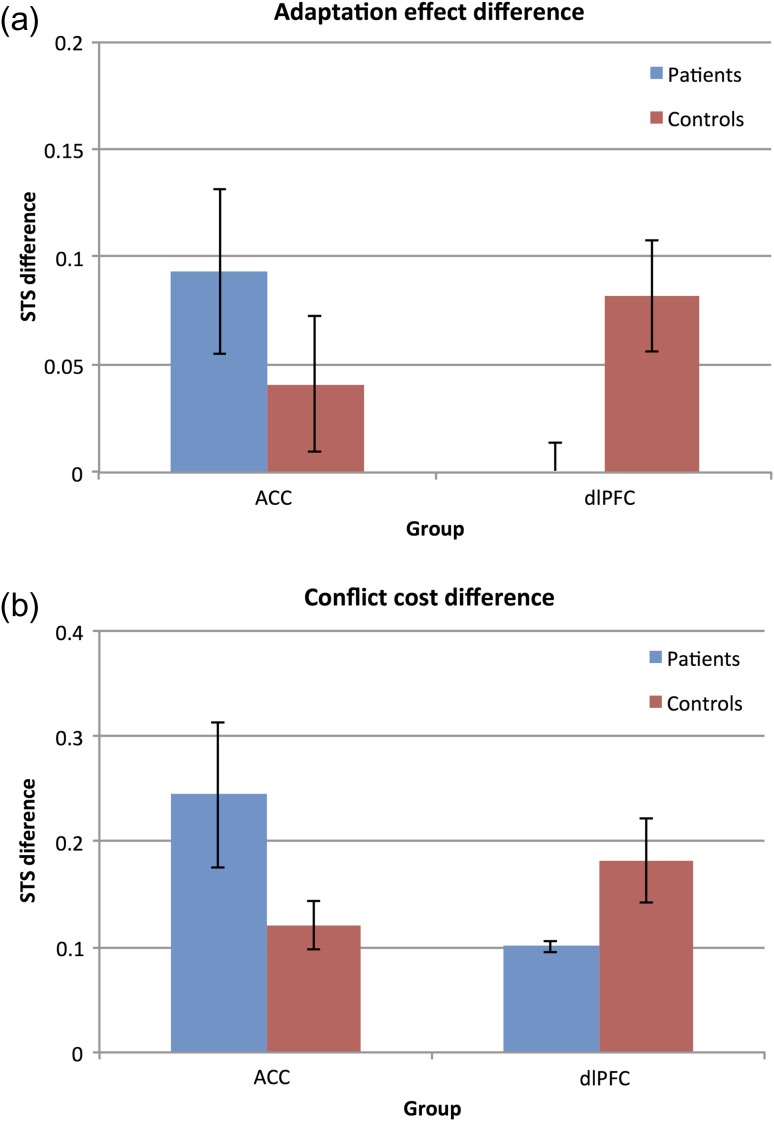
STS on different trial types across groups, (*a*) Mean STS difference between HH and LH trials (i.e. adaptation effect) for each patient group plotted against their respective matched control group. The presence of an adaptation effect (i.e. a positive STS difference score) indicates faster responses on HH trials compared with LH trials. (*b*) Mean STS difference between low- and high-conflict trials (i.e. conflict effect) for each patient group plotted against their respective matched control group. The presence of a conflict effect (i.e. a positive STS difference score) indicates faster responses on low-conflict compared with high-conflict trials.

**Figure 5.**
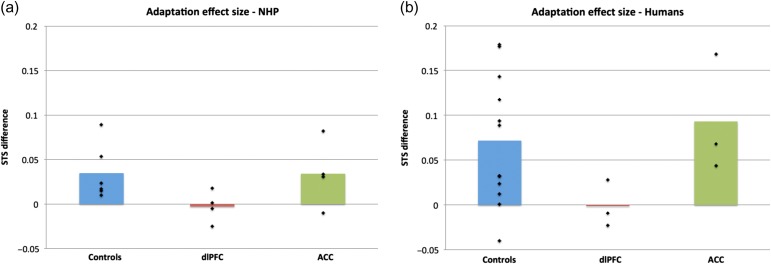
Effect of prefrontal lesions on behavioral adaptation in nonhuman and human primates. Mean normalized STS difference between HH and LH trials (i.e. adaptation effect) indicated by bars, and individual normalized STS indicated by black markers for nonhuman primate control and lesion groups in panel (*a*) [data adapted with permission from Mansouri et al. 2007] and human control and lesion groups in panel (*b*). In both nonhuman and human primates, lesions to dlPFC, but not ACC abolish the adaptation effect.

Firstly, with regard to our conflict-induced behavioral adaptation measure, as Mansouri et al. (2007) indicated a clear difference in adaptation between the dlPFC lesioned macaques and unoperated controls, but not between the ACC lesioned macaques and unoperated controls, we predicted that there may be a similar difference in the same adaptation measure in our dlPFC patients compared with their matched control group but not in our ACC group compared with their matched control group. To test whether dlPFC patients also showed reduced adaptation, as previously shown in monkeys, we used a one-tailed *t*-test. As hypothesized, a Mann–Whitney test for independent samples indicated a significant difference in the adaptation effect between the dlPFC group and their control group (*z* = −1.81, *P* = 0.035, one-tailed test), with the control group showing significantly larger STS difference between HH and LH than the dlPFC group. Further consistent with our hypothesis, there was no significant difference in the size of the adaptation effect between the ACC group and their controls (*z* = −1.29, *P* = 0.197, 2-tailed test). These results are illustrated in Figure 4*a* and replicate the effects previously observed in nonhuman primates with dlPFC lesions on the same task and using the same measures (Mansouri et al. 2007, see Fig. 5).

Secondly, with regard to our conflict-cost measure, CMCC theory would predict that ACC patients should show significantly larger conflict-costs compared with their control group, whereas dlPFC patients should show similar conflict-costs to their control group. However, inconsistent with CMCC theory, Mann–Whitney tests for independent samples revealed no significant differences in the size of conflict-cost effects between either of the patient groups and their corresponding matched controls: ACC patients did not significantly differ from their controls on the conflict-cost measure (*z* = −1.55, *P* = 0.121, 2-tailed test), and neither did the dlFPC patients compared with their controls (*z* = −1.29, *P* = 0.197, 2-tailed test) (Fig. 4*b*).

## Discussion

In this study, we aimed to investigate the contributions of human ACC and dlPFC toward conflict monitoring and conflict-induced behavioral adaptation using a well-established version of the macaque WCST analog (Mansouri et al. 2007, 2009, 2014, 2015; Kuwabara et al. 2014) that elicits conflict between 2 task-specific responses. Replicating Mansouri et al.’s (2007) findings in nonhuman primates, our study confirmed that one frontal area that does affect patients’ abilities to adjust their behavior on H trials as a function of previous trial type is dlPFC. Patients with dlPFC lesions exhibited a significantly reduced difference, compared with controls, between H trials preceded by H trials (HH) and H trials preceded by L trials (LH). This result is consistent with the aforementioned nonhuman primate findings (Mansouri et al. 2007) and also with a body of literature implicating the dlPFC as an important structure for behavioral adaptation in the presence of conflict (Durston et al. 2003; Kerns et al. 2004; Egner and Hirsch 2005; Kerns 2006; Kim et al. 2012). Our findings also indicate that patients whose lesions include the ACC are not impaired in our measure of conflict-cost (i.e. the differential is their slower speed of responding to high-conflict (H) trials versus their faster speed of responding to low-conflict (L) trials), nor in our measures of conflict-induced behavioral adaptation (i.e. the differential is their slower speed of responding to H trials after L trials vs. their faster speed of responding to H trials after H trials) as compared with their matched controls. This confirms that the human ACC, like nonhuman primate ACC (Mansouri et al. 2007), is not generally necessary for optimal performance in the presence of conflict, when this is elicited between 2 abstract, task-specific responses.

The absence of impairment in the ACC group on H trials (i.e. a conflict-detection/conflict-cost effect) is perhaps unsurprising when one takes overall account of the neuropsychological evidence on a range of tasks eliciting conflict. Most of the human lesion studies that investigated the role of the ACC in responding to H trials and subsequent adaptation found either no impairment (Vendrell et al. 1995; Stuss et al. 2001; Swick and Jovanovic 2002; Fellows and Farah 2005) or impairment restricted to the subsequent adaptation (di Pellegrino et al. 2007; Sheth et al. 2012). Di Pellegrino et al. (2007) found that the conflict cost in a Simon task was significantly larger in the patient groups than in the control group, however, there was no significant difference among patient groups regardless of the specific region affected by the lesion; instead they found that only adaptation was affected specifically by ACC damage. These findings were replicated in a study on cingulotomy patients (Sheth et al. 2012). This indicates that while ACC activity is sensitive to the level of conflict in the task, as demonstrated by neuroimaging, it is not a crucial substrate for aiding conflict resolution on the current trial and/or that the areas that may be involved in conflict resolution on the current trial are not dependent on ACC alone for their performance (see Carter and van Veen 2000). However, our ACC patients were also not impaired on measures of conflict-induced behavioral adaptation. While this parallels the data from the nonhuman primate study with highly circumscribed lesions (Mansouri et al. 2007), both studies remain at odds with the human literature showing impairments in adaptation following ACC damage (Swick and Turken 2002; Di Pellegrino et al. 2007; Sheth et al. 2012).

One explanation for these discrepancies that is already a focus of argument and counter-argument in the literature concerns whether ACC damage extends into cingulofrontal transition area 32′, which has been proposed as the specific ACC subregion potentially responsible for conflict monitoring in humans (see Cole et al. 2009, 2010; Schall and Emeric 2010). Cole et al. (2009) have highlighted anatomical evidence (Vogt et al. 1995) that area 32′ may even be unique to humans and have explicitly suggested that “both monkeys and humans monitor for motor conflict (area 24′), whereas only humans monitor for non-motor decision conflict (area 32′).”

Considering the extent of our patients’ lesions in this light, it is apparent that in our ACC cohort, only a very limited amount of the lesions encroached onto area 32′. In fact, while ACC damage “across” participants covered a vast proportion of this region, each of our 3 patients presented lesions to different, nonoverlapping, ACC subregions other than 32′. Damage encompassed instead mostly areas 32 and 24 and it is, therefore, possible that the lack of impairment in measures of conflict monitoring or adaptation in our population is due to the fact that none of the affected subregions are recruited for processing nonmotor decision conflict in humans, as suggested by Cole et al. (2009).

However, there are a number of issues with this interpretation. First, other authors suggest area 32′, while more extensive in humans, may be homologous to a less extensive region within the macaque cingulate motor areas (Ongür et al. 2003; Schall and Emeric 2010), and that the discrepancy between results from human and monkeys, indicating an ACC role in conflict monitoring in the former but not the latter, is actually due to differences in effectors. Human studies generally involve forelimb movements, which might be more strongly dependent on the ACC, while monkey studies generally involve eye-movements, which might be more strongly dependent on the supplementary eye-fields (Schall and Emeric 2010). Indeed, just as studies using eye-movements found conflict-sensitive neurons in the supplementary eye-fields (Nakamura et al. 2005), more recent electrophysiological studies that used forelimb movements in monkeys succeeded in finding conflict-sensitive neurons in the monkey cingulate motor area (Michelet et al. 2016). We agree that it is crucial to consider effector systems and, indeed, a key aim of this study was to bring a direct comparison with Mansouri et al. (2007) to reveal that similar behavioral deficits can occur after dlPFC but not after ACC lesions in both humans and monkeys when the same effector system is employed (i.e. forelimb movement to a touchscreen). The marked similarity in the results obtained from this study and Mansouri and colleagues nonhuman primate study helps inform the debate regarding similarities or differences between human and monkey PFC contributions to cognition and demonstrates that despite the differences there can be great value in cross-species neuropsychological comparisons using the same behavioral tasks. In addition, other studies investigating the effects of fairly large ACC lesions (including 32′) in human patients have similarly failed to find an effect of ACC damage on conflict monitoring and adaptation (Fellows and Farah 2005), suggesting that such findings are not exclusive to nonhuman primate studies.

Secondly, this interpretation depends upon classification of our WCST analog as an exclusively nonmotor decision conflict task, as opposed to a task that might present also motor conflict. While we consider our task to primarily elicit conflict at the level of the nonmotor decision between 2 abstract rules (because the correct motor response is randomized from trial-to-trial in the lesion study WCST conflict task), the effector system in the WCST conflict task involves forelimb movement and hence one could reason that conflict may also appear at that level. If subdivisions of the ACC other than 32′ are devoted to resolving motor-related conflict, then we would expect to observe a certain degree of impairment on the task in our patient population as a result of lesions to these more caudal and dorsal cingulate subdivisions.

Therefore, anatomical discrepancies between species and the specific location of the damage in our patient population are unlikely to fully account for why our findings appear to contradict the CMCC model. We believe a better explanation for our results might be related to the specific features of the task used. As already emphasized, the WCST conflict analog used in our study is designed to elicit conflict between 2 task-relevant abstract rules, in contrast with other conflict tasks that elicit conflict between task-relevant responses and more habitual task-irrelevant responses.

Tasks commonly used to investigate conflict monitoring (e.g. Simon, Flanker, and Stroop) include L trials that do not require any specific rule to be retrieved in order to perform the correct response and, further, may exploit a habitual and/or overpracticed task-irrelevant response (e.g. word-reading or responding to the location of a stimulus) to speed up responses. In contrast, in the WCST analog there is no such habitual response that can aid performance on L trials, as there is no task-irrelevant predisposition to select the color-match over the shape-match or vice versa. Therefore, while in “classic” conflict tasks, H trials are characterized by competition between the task-relevant response and a task-irrelevant habit, in the WCST conflict is elicited exclusively between 2 task-specific responses. This may account for why conflict-monitoring studies that included neutral trials (i.e. trials that do not involve an element of facilitation due to the exploitation of a habit) in their tasks found neutral trials to be generally slower than L trials (Milham et al. 2001; Wühr and Ansorge 2005; Galashan et al. 2008; Chen and Melara 2009). Higher ACC activation was indeed reported for L and H trials than for neutral trials (Milham and Banich 2005).

Indeed, with regards to the ongoing debate about whether or not monkeys perform conflict monitoring in a manner similar to humans and, if so, whether or not monkey ACC is crucial for this process, new electrophysiology studies in the macaque monkey have shown that conflict-sensitive neurons are indeed observed in the monkey ACC when the task is designed in such a way as to elicit competition between task-relevant information and task-irrelevant information that is highly salient to the animal in nonexperimental, naturalistic settings (Ebitz and Platt 2015), or when conflict is elicited between a currently relevant response and an overpracticed association (Michelet et al. 2016). Ebitz and Platt's (2015) study in particular addresses ideas that share several commonalities with those we propose here. Namely, their distinction between conflict elicited at the level of physical actions within the task (i.e. “action conflict”) and conflict elicited at the level of task set (i.e. “task conflict”) can be seen as analog to our distinction between conflict elicited between task-specific instructed responses and conflict elicited between task-specific responses and task-irrelevant habitual responses, respectively. These authors characterized “task conflict” by using biologically salient distractors (i.e. monkey faces) to compete with nonbiologically salient stimuli (i.e. a target square), whereas “action conflict” was characterized by using neutral distractors (i.e. phase-scrambled versions of the monkey faces stimuli) to compete with the target. ACC neurons were found to respond more robustly and consistently when competition was elicited between the biologically salient distractors and the target than when it was elicited between the neutral distractors and the target. The biological salience of the distractors in the “task conflict” condition in Ebitz and Platt's monkey task can be seen as similar concept to that of pre-established, task-irrelevant habitual responses we suggest for human conflict tasks. In both cases (monkeys and humans), the distractor or competing response that needs to be suppressed/ignored in favor of the task-relevant target or response is highly salient and/or primed in naturalistic settings relevant to either species (e.g. other monkeys for nonhuman primates, and word-reading for humans). It is, therefore, reasonable to conclude that conflict-sensitive neurons had not yet been detected in nonhuman primates before the Ebitz and Platt's (2015) study because the paradigms used in the past had not tapped into the crucial distinction between task-relevance versus naturalistic salience. Consistently, as the conflict WCST analog does not involve any conflict between task-relevant versus naturalistic salient responses, it would not be expected to rely on an intact ACC for optimal performance.

All these findings suggest that human and nonhuman primate ACC is not a neural substrate necessary for supporting “general” conflict-detection or signaling adaptation to conflict (defined as competition between any 2 or more responses) but, rather, might be important only in specific circumstances where conflict is elicited between a task-relevant response and a pre-existing habit as it is the case in more commonly used conflict paradigms.

While this interpretation may be at odds with the classic CMCC model of ACC function, it is largely consistent with more recent frameworks suggesting a role for ACC in outcome evaluation in order to support learning (Silvetti et al. 2014), particularly in conditions of varying uncertainty (Behrens et al. 2007; Rushworth and Behrens 2008), and in flexible decision-making in contexts that require the selection of options that deviate from previous best long-term options (Boorman et al. 2013).

There is a large body of evidence describing ACC's involvement in monitoring and tracking the outcome of actions, both negative—that is errors (Braver et al. 2001; Ito et al. 2003; Holroyd et al. 2005; Wang et al. 2005; Brown and Braver 2008; West and Travers 2008; Hayden et al. 2009; Nee et al. 2010; Neta et al. 2014, 2016)—and positive—that is rewards (Ito et al. 2003; Kennerley et al. 2006, 2011; Matsumoto et al. 2007; Emeric et al. 2008; Rudebeck et al. 2008).

Optimal feedback processing is crucial to support behavior in contexts where the uncertainty regarding the appropriate course of action is increased. The presence of conflict between responses indeed represents one such context. However, there are a wide variety of other such contexts that have also been consistently associated with ACC's involvement, such as cognitively demanding tasks (Davis et al. 2000; Davis and Taylor 2005; Silton et al. 2010), the presence of novel, surprising and unexpected or rare events (Braver et al. 2001; Ito et al. 2003; Badre and Wagner 2004; Nee et al. 2010; Hayden et al. 2011; Wilk et al. 2012), and environmental volatility or ambiguity (Ridderinkhof and Ullsperger 2004; Behrens et al. 2007; Neta et al. 2014). Given ACC's recruitment in a wide variety of different experimental conditions, all characterized by uncertainty, we believe that a more parsimonious explanation for ACC's role in conflict tasks can be framed in terms of a more general monitoring function concerned with feedback processing in situations that require the identification of the most advantageous response given the current context and, consequently, flexible adjustments in behavior, rather than to strictly perform conflict monitoring.

Accordingly, ACC should be far more crucial in a conflict task requiring a switch in behavior away from best long-term options (see Boorman et al. 2013), which habitual or overpracticed responses can represent, in favor of a different response that is advantageous specifically within the current task demands (or even trial demands), rather than in a task where the switch occurs between 2 equally rewarded, task-specific rules, as in the case of our study. That is because the former type of task would present a much higher degree of uncertainty and error likelihood than the latter, and therefore place higher demands on feedback tracking and action evaluation for optimal performance. Lesion studies in the macaque monkey have indeed already demonstrated that animals with lesions to the ACC are unable to optimally use information about sustained recent positive feedback to identify new advantageous responses that are to be selected over previously advantageous ones (Kennerley et al. 2006).

One current area of debate concerns whether ACC's signaling in neuroimaging studies using conflict tasks is related to conflict monitoring or time on task (Brown 2011; Grinband et al. 2011; Yeung et al. 2011; Weissman and Carp 2013), with recent evidence supporting the latter interpretation over the conflict-monitoring view (Weissman and Carp 2013). The outcome evaluation framework of ACC function can be seen as largely consistent with the time on task account. The degree of uncertainty about the best behavioral option in a given context would obviously be expected to modulate RTs; the higher the uncertainty regarding the correct response, the more time required by the cognitive system to evaluate all potential responses and accumulate the evidence necessary to select the most appropriate one. This, neurobiologically, would be expected to translate into increased demand on the ACC, and therefore should result in correlations between RT and ACC signaling.

Therefore, ACC activation in neuroimaging studies using conflict tasks such as Stroop or Simon tasks might be highlighting processes due to the need to track and evaluate outcomes in order to prime deviations from habitual responses, rather than general conflict-monitoring mechanisms. Indeed, studies that have investigated the effects of practice on performance in conflict tasks found that, while behavioral measures of conflict remained fairly consistent throughout, ACC activity decreased as the task became overpracticed (Erickson et al. 2004), often quite rapidly (Milham et al. 2003), as the participants became more and more conditioned to respond to high-conflict trials. In other words, the more habituated the type of response, and thus the lower uncertainty and the demands on action evaluation processes, the less ACC is recruited, despite conflict still being present (as evidenced by the behavioral measures).

This interpretation could also help explain the findings from human patients that show no impairment on conflict-induced adaptation following ACC damage. For example, Stuss et al. (2001) found no effects of ACC damage on performance on the incongruent trials in the Stroop task. However, they only administered patients with the incongruent version of the task. If participants are only exposed to incongruent trials, they might automatize color-naming (as opposed to word-reading), as this is the only response that can be rewarded during the task. Therefore, the competition between responses, degree of uncertainty and demands on the evaluative processes are significantly reduced in this context. Similarly, Fellows and Farah (2005), also in a Stroop task, found no effect of ACC damage on the ability of participants to adjust their behavior depending on the percentage of high-conflict trials. However, they administered participants with the mostly high-conflict block first, and only later with the mostly low-conflict block. Again, this might have encouraged participants to automatize the color-naming response during the first block, therefore reducing the effects of competition between the habitual word-reading response and the instructed color-naming response in the subsequent block.

While our ACC findings call for a re-interpretation of its role within the conflict-monitoring framework, the findings of impairments in adaptation after dlPFC lesions are largely consistent with the imaging literature on this topic (Durston et al. 2003; Kerns et al. 2004; Egner and Hirsch 2005; Kerns 2006; Kim et al. 2012). Human neuropsychological evidence on the necessity of an intact dlPFC to perform optimal behavioral adaptation in the presence of conflict is still very scarce, but transcranial magnetic stimulation to this region has been shown to impair adaptation in the Simon task (Stürmer et al. 2007). What our findings suggest, further to the existing literature, is that dlPFC, unlike ACC, appears to be crucial for behavioral adaptation regardless of the manner in which conflict is elicited (i.e. between a habitual and task-specific response, as in the aforementioned cases, or between task-specific responses). This implies that, despite the frequent coactivation of these 2 regions in conflict tasks, ACC, and dlPFC may play different roles in terms of the specific mechanisms they support that contribute to adaptation.

Both dlPFC and ACC have been characterized as components of 2 separate cortical networks involved in cognitive control, with the former belonging to a frontoparietal network and the latter to a cingulopercular network (Dosenbach et al. 2007). These 2 networks have been suggested to be functionally distinct, with the frontoparietal network being involved in the accumulation and maintenance of evidence and control signals online across a small number of trials (supporting the implementation of task control from trial to trial), and the cingulopercular network being involved in monitoring and tracking of the outcome of decisions over longer timescales, supporting a more “stable” form of task control (Dosenbach et al. 2007; Neta et al. 2015; Gratton et al. 2016).

This proposed functional distinction is consistent with the idea of ACC being involved in the continuous evaluation of action outcomes in order to determine the best behavioral option in a given context, and particularly in determining when it is necessary to deviate from a previously preferred (and therefore previously “stable”) option, while dlPFC might be concerned with keeping track of trial-to-trial contextual changes. Evidence from electrophysiological recordings in macaque monkeys performing the conflict WCST analog have indeed shown that dlPFC neurons appear to maintain information about the conflict level of a trial (both LH and HH trials) during the intertrial period (Mansouri et al. 2007). Furthermore, neuroimaging studies looking at sequences of several trials have shown that dlPFC activity on high-conflict trials increases as a function of the number of preceding consecutive low-conflict trials (i.e. LLH, LLLH, LLLLH, and so on) and decrease as a function of the number of preceding consecutive high-conflict trials (Durston et al. 2003). This suggests that, in conflict tasks, dlPFC is not recruited more generally in the presence of sustained high-conflict, but more specifically during narrow time-windows centered on time points that signal changes in the level of conflict from one trial to the next (e.g. from low to high conflict—LH trials, and shortly thereafter—HH trials). This is consistent with the idea of its role in a frontoparietal network concerned with maintaining information online across relatively short timescales to aid task control. This interpretation would also explain why dlPFC is crucial for both conflict tasks involving competition between habitual and task-specific responses and conflict tasks involving competition between task-specific responses only, as it would be concerned with maintaining information about conflict “history,” rather than the nature of the conflict per se, or with evaluating specific responses against one another. Most conflict-monitoring studies to date have varied the levels of conflict randomly throughout the task. However, future studies manipulating the specific sequence of H and L trials, and particularly the “volatility” of conflict levels within a task (i.e. how often they change from low to high and vice versa), could be particularly useful to shed more light on the specific time points at which ACC and dlPFC are recruited, which could in turn help discern their distinct functional contributions to behavioral adaptation.

One significant limitation of our study lies in the lack of lesion specificity and the little to no overlap in lesion location across patients, particularly in our ACC group. This is an unfortunately common obstacle in human neuropsychological investigations. We nonetheless believe that our results (particularly to the extent that they complement and replicate Mansouri et al.’s (2007) findings), have begun to unveil an important aspect of conflict-monitoring frameworks, more specifically, and of ACC function, more broadly. Thus, it would be extremely valuable for future research to probe further into the issue of task-specific versus habitual responses in order to verify whether our findings can be reliably be replicated in the presence of more targeted or more overlapping lesions in human patient populations.

To conclude, in a task eliciting conflict between 2 task-specific responses that did not involve suppression of a habitual response, lesions to dlPFC affect adaptation, by abolishing the adaptation effect in patients compared with controls. On the other hand, lesions to ACC do not affect either conflict-costs or conflict-induced adaptation on the current trial as a function of the level of conflict on the previous trial. These findings suggest that while the CMCC model might provide an account for some aspects of conflict monitoring and adaptation (e.g. dlPFC playing an important role in adaptation), it remains necessary to better elucidate the specific contributions of ACC in modulating behavior in response to different types of conflict and the distinct cognitive processes that may underlie adaptation.

## Supplementary Material

Supplementary material can be found here.

## Author Contributions

M.J.B. designed the research; E.A.B., M.M.B., and M.J.B. conducted the research; E.A.B. and M.J.B. analyzed the data and prepared the manuscript; J.S.S. helped facilitate recruitment of volunteers, advised on the analyses and helped with manuscript preparation.

## Supplementary Material

Supplementary Data

## Notes

We wish to thank the MRC Cognition and Brain Sciences Unit, Cambridge, for their help and support in the recruitment of volunteers for this study and access to the facilities, particularly P. Peers and R. Dunn. We also wish to thank G. Humphreys and N. Demeyere for access to the volunteers panel at the University of Birmingham. *Conflict of Interest*: None declared.

## References

[bhw350C1] Badre D, Wagner AD 2004 Selection, integration, and conflict monitoring; assessing the nature and generality of prefrontal cognitive control mechanisms. Neuron. 41(3):473–487. 14766185 10.1016/s0896-6273(03)00851-1

[bhw350C2] Barch DM, Braver TS, Sabb FW, Noll DC 2000 Anterior cingulate and the monitoring of response conflict: evidence from an fMRI study of overt verb generation. J Cogn Neurosci. 12(2):298–309. 10.1162/089892900562110. 10771413

[bhw350C3] Behrens TEJ, Woolrich MW, Walton ME, Rushworth MFS 2007 Learning the value of information in an uncertain world. Nat Neurosci. 10(9):1214–1221. 10.1038/nn1954. 17676057

[bhw350C4] Boorman ED, Rushworth MF, Behrens TE 2013 Ventromedial prefrontal and anterior cingulate cortex adopt choice and default reference frames during sequential multi-alternative choice. J Neurosci. 33(6):2242–2253. 10.1523/JNEUROSCI.3022-12.2013. 23392656 PMC3743024

[bhw350C5] Botvinick M 2007 Conflict monitoring and decision making: reconciling two perspectives on anterior cingulate function. Cogn Affect Behav Neurosci. 7(4):356–366. 18189009 10.3758/cabn.7.4.356

[bhw350C6] Botvinick M, Braver T, Barch D 2001 Conflict monitoring and cognitive control. Psychol Rev. 108:624–652. 11488380 10.1037/0033-295x.108.3.624

[bhw350C7] Braver TS, Barch DM, Gray JR, Molfese DL, Snyder A 2001 Anterior cingulate cortex and response conflict: effects of frequency, inhibition and errors. Cereb Cortex. 11(9):825–836. 11532888 10.1093/cercor/11.9.825

[bhw350C8] Brown JW 2011 Medial prefrontal cortex activity correlates with time-on-task: what does this tell us about theories of cognitive control. NeuroImage. 57(2):314–315. 10.1016/j.neuroimage.2011.04.028. 21540116

[bhw350C9] Brown JW, Braver TS 2008 A computational model of risk, conflict, and individual difference effects in the anterior cingulate cortex. Brain Res. 1202:99–108. 10.1016/j.brainres.2007.06.080. 17707352 PMC2322871

[bhw350C10] Buchsbaum BR, Greer S, Chang W-L, Berman KF 2005 Meta-analysis of neuroimaging studies of the Wisconsin card-sorting task and component processes. Hum Brain Mapp. 25(1):35–45. 10.1002/hbm.20128. 15846821 PMC6871753

[bhw350C11] Carter C, van Veen V 2007 Anterior cingulate cortex and conflict detection: an update of theory and data. Cogn Affect Behav Neurosci. 7(4):367–379. 18189010 10.3758/cabn.7.4.367

[bhw350C93] Chen S, Melara RD 2009 Sequential effects in the Simon task: conflict adaptation or feature integration. Brain Res. 1297:89–100. 10.1016/j.brainres.2009.08.003. 19666010

[bhw350C12] Chen Q, Wei P, Zhou X 2006 Distinct neural correlates for resolving stroop conflict at inhibited and noninhibited locations in inhibition of return. J Cogn Neurosci. 18(11):1937–1946. 10.1162/jocn.2006.18.11.1937. 17069483

[bhw350C13] Cohen RA, Kaplan RF, Moser DJ, Jenkins MA, Wilkinson H 1999 Impairments of attention after cingulotomy. Neurology. 53(4):819–819. 10.1212/WNL.53.4.819. 10489048

[bhw350C14] Cole MW, Yeung N, Freiwald WA, Botvinick M 2010 Conflict over cingulate cortex: between-species differences in cingulate may support enhanced cognitive flexibility in humans. Brain Behav Evol. 75(4):239–240. 10.1159/000313860. 20693782

[bhw350C15] Cole MW, Yeung N, Freiwald WA, Botvinick M 2009 Cingulate cortex: diverging data from humans and monkeys. Trends Neurosci. 32(11):566–574. 10.1016/j.tins.2009.07.001. 19781794 PMC7580873

[bhw350C16] Davis KD, Hutchison WD, Lozano AM, Tasker RR, Dostrovsky JO 2000 Human anterior cingulate cortex neurons modulated by attention-demanding tasks. J Neurophysiol. 83(6):3575–3577. 10848573 10.1152/jn.2000.83.6.3575

[bhw350C17] Davis K, Taylor K 2005 Human anterior cingulate cortex neurons encode cognitive and emotional demands. J Neurosci. 25:8402–8406. 16162922 10.1523/JNEUROSCI.2315-05.2005 PMC6725669

[bhw350C18] di Pellegrino G, Ciaramelli E, Làdavas E 2007 The regulation of cognitive control following rostral anterior cingulate cortex lesion in humans. J Cogn Neurosci. 19(2):275–286. 10.1162/jocn.2007.19.2.275. 17280516

[bhw350C19] Dosenbach NUF, Fair DA, Miezin FM, Cohen AL, Wenger KK, Dosenbach RAT, Fox MD, Snyder AZ, Vincent JL, Raichle ME, et al 2007 Distinct brain networks for adaptive and stable task control in humans. Proc Natl Acad Sci U S A. 104(26):11073–11078. 10.1073/pnas.0704320104. 17576922 PMC1904171

[bhw350C20] Durston S, Davidson M, Thomas K, Worden M, Tottenham N, Martinez A, Watts R, Ulug AM, Casey B 2003 Parametric manipulation of conflict and response competition using rapid mixed-trial event-related fMRI. NeuroImage. 20(4):2135–2141. 10.1016/j.neuroimage.2003.08.004. 14683717

[bhw350C21] Ebitz RB, Platt ML 2015 Neuronal activity in primate dorsal anterior cingulate cortex signals task conflict and predicts adjustments in pupil-linked arousal. Neuron. 85(3):628–640. 10.1016/j.neuron.2014.12.053. 25654259 PMC4319115

[bhw350C22] Egner T, Hirsch J 2005 The neural correlates and functional integration of cognitive control in a stroop task. NeuroImage. 24(2):539–547. 10.1016/j.neuroimage.2004.09.007. 15627596

[bhw350C23] Emeric EE, Brown JW, Leslie M, Pouget P, Stuphorn V, Schall JD 2008 Performance monitoring local field potentials in the medial frontal cortex of primates: anterior cingulate cortex. J Neurophysiol. 99(2):759–772. 10.1152/jn.00896.2006. 18077665 PMC2675936

[bhw350C24] Erickson KI, Milham MP, Colcombe SJ, Kramer AF, Banich MT, Webb A, Cohen NJ 2004 Behavioral conflict, anterior cingulate cortex, and experiment duration: implications of diverging data. Hum Brain Mapp. 21(2):98–107. 10.1002/hbm.10158. 14755597 PMC6871972

[bhw350C25] Fan J 2003 Cognitive and brain consequences of conflict . NeuroImage. 18(1):42–57. 10.1006/nimg.2002.1319. 12507442

[bhw350C26] Fellows LK, Farah MJ 2005 Is anterior cingulate cortex necessary for cognitive control. Brain. 128:788–796. 15705613 10.1093/brain/awh405

[bhw350C90] Galashan D, Wittfoth M, Fehr T, Herrmann M 2008 Two Simon tasks with different sources of conflict: an ERP study of motion- and location-based compatibility effects. Biol Psychol. 78 (3):246–52. 10.1016/j.biopsycho.2008.03.008. 18436365

[bhw350C91] Grant D, Berg E 1948 A behavioural analysis of reinforcement and ease of shifting to new responses in a Weigl-type card-sorting problem. J Exp Psychol. 404–411. 18874598 10.1037/h0059831

[bhw350C27] Gratton C, Neta M, Sun H, Ploran EJ, Schlaggar BL, Wheeler ME, Petersen SE, Nelson SM 2016 Distinct stages of moment-to-moment processing in the cinguloopercular and frontoparietal networks. Cereb Cortex. doi:10.1093/cercor/bhw092. 10.1093/cercor/bhw092 PMC6411212 27095824

[bhw350C28] Grinband J, Savitskaya J, Wager TD, Teichert T, Ferrera VP, Hirsch J 2011 Conflict, error likelihood, and RT: response to Brown & Yeung et al. NeuroImage. 57(2):320–322. 10.1016/j.neuroimage.2011.04.027. 21554960 PMC5325718

[bhw350C29] Hayden BY, Heilbronner SR, Pearson JM, Platt ML 2011 Surprise signals in anterior cingulate cortex: neuronal encoding of unsigned reward prediction errors driving adjustment in behavior. J Neurosci. 31(11):4178–4187. 10.1523/JNEUROSCI.4652-10.2011. 21411658 PMC3070460

[bhw350C30] Hayden BY, Pearson JM, Platt ML 2009 Fictive reward signals in the anterior cingulate cortex. Science. 324(5929):948–950. 10.1126/science.1168488. 19443783 PMC3096846

[bhw350C31] Hazeltine E 2003 Material-dependent and material-independent selection processes in the frontal and parietal lobes: an event-related fMRI investigation of response competition. Neuropsychologia. 41(9):1208–1217. 10.1016/S0028-3932(03)00040-X. 12753960

[bhw350C32] Hedge A, Marsh N 1975 The effect of irrelevant spatial correspondences on two-choice response-time. Acta Psychol (Amst). 39(6):427–439. 1199779 10.1016/0001-6918(75)90041-4

[bhw350C33] Holroyd CB, Yeung N, Coles MGH, Cohen JD 2005 A mechanism for error detection in speeded response time tasks. J Exp Psychol Gen. 134(2):163–191. 10.1037/0096-3445.134.2.163. 15869344

[bhw350C34] Ito S, Stuphorn V, Brown JW, Schall JD 2003 Performance monitoring by the anterior cingulate cortex during saccade countermanding. Science. 302(5642):120–122. 10.1126/science.1087847. 14526085

[bhw350C35] Kennerley SW, Behrens TEJ, Wallis JD 2011 Double dissociation of value computations in orbitofrontal and anterior cingulate neurons. Nat Neurosci. 14(12):1581–1589. 10.1038/nn.2961. 22037498 PMC3225689

[bhw350C36] Kennerley SW, Walton ME, Behrens TEJ, Buckley MJ, Rushworth MFS 2006 Optimal decision making and the anterior cingulate cortex. Nat Neurosci. 9(7):940–947. 10.1038/nn1724. 16783368

[bhw350C37] Kerns JG 2006 Anterior cingulate and prefrontal cortex activity in an FMRI study of trial-to-trial adjustments on the Simon task. NeuroImage. 33(1):399–405. 10.1016/j.neuroimage.2006.06.012. 16876434

[bhw350C98] Kerns JG, Cohen JD, MacDonald AW, Cho RY, Stenger VA, Carter CS 2004 Anterior cingulate conflict monitoring and adjustments in control. Science. 303 (5660):1023–6. 10.1126/science.1089910. 14963333

[bhw350C38] Kim C, Chung C, Kim J 2012 Conflict adjustment through domain-specific multiple cognitive control mechanisms. Brain Res. 1444:55–64. 10.1016/j.brainres.2012.01.023. 22305142

[bhw350C39] Kim C, Kroger JK, Kim J 2011 A functional dissociation of conflict processing within anterior cingulate cortex. Hum Brain Mapp. 32(2):304–312. 10.1002/hbm.21020. 21229616 PMC6869912

[bhw350C40] Kornblum S, Hasbroucq T, Osman A 1990 Dimensional overlap: cognitive basis for stimulus-response compatibility–a model and taxonomy. Psychol Rev. 97(2):253. 2186425 10.1037/0033-295x.97.2.253

[bhw350C41] Kuwabara M, Mansouri FA, Buckley MJ, Tanaka K 2014 Cognitive control functions of anterior cingulate cortex in macaque monkeys performing a Wisconsin Card Sorting Test analog. TJ Neurosci. 34(22):7531–7547. 10.1523/JNEUROSCI.3405-13.2014. PMC4035517 24872558

[bhw350C42] Lie C-H, Specht K, Marshall JC, Fink GR 2006 Using fMRI to decompose the neural processes underlying the Wisconsin Card Sorting Test. NeuroImage. 30(3):1038–1049. 10.1016/j.neuroimage.2005.10.031. 16414280

[bhw350C43] Lu C, Proctor RW 1995 The influence of irrelevant location information on performance : a review of the Simon and spatial Stroop effects. Psychon Bull Rev. 2(2):174–207. 24203654 10.3758/BF03210959

[bhw350C44] MacDonald AW 2000 Dissociating the role of the dorsolateral prefrontal and anterior cingulate cortex in cognitive control. Science. 288(5472):1835–1838. 10.1126/science.288.5472.1835. 10846167

[bhw350C45] Mansouri FA, Buckley MJ, Tanaka K 2007 Mnemonic function of the dorsolateral prefrontal cortex in conflict-induced behavioral adjustment. Science. 318(5852):987–990. 10.1126/science.1146384. 17962523

[bhw350C46] Mansouri FA, Buckley MJ, Tanaka K 2014 The essential role of primate orbitofrontal cortex in conflict-induced executive control adjustment. J Neurosci. 34(33):11016–11031. 10.1523/JNEUROSCI.1637-14.2014. 25122901 PMC4131015

[bhw350C47] Mansouri FA, Tanaka K, Buckley MJ 2009 Conflict-induced behavioural adjustment: a clue to the executive functions of the prefrontal cortex. Nat Rev Neurosci. 10(2):141–152. 10.1038/nrn2538. 19153577

[bhw350C48] Mansouri f. A., Buckley MJ, Mahboubi M, Tanaka K 2015 Behavioral consequences of selective damage to frontal pole and posterior cingulate cortices. Proc Natl Acad Sci. 10.1073/pnas.1422629112, 201422629. PMC4517212 26150522

[bhw350C49] Matsumoto M, Matsumoto K, Abe H, Tanaka K 2007 Medial prefrontal cell activity signaling prediction errors of action values. Nat Neurosci. 10(5):647–656. 10.1038/nn1890. 17450137

[bhw350C50] Michelet T, Bioulac B, Langbour N, Goillandeau M, Guehl D, Burbaud P 2016 Electrophysiological correlates of a versatile executive control system in the monkey anterior cingulate cortex. Cereb Cortex. 26(4):1684–1697. 10.1093/cercor/bhv004. 25631057

[bhw350C102] Milham MP, Banich MT, Webb A, Barad V, Cohen NJ, Wszalek T, Kramer AF 2001 The relative involvement of anterior cingulate and prefrontal cortex in attentional control depends on nature of conflict. Brain Res Cogn Brain Res. 12 (3):467–73. 11689307 10.1016/s0926-6410(01)00076-3

[bhw350C51] Milham M, Banich MT, Claus E, Cohen N 2003 Practice-related effects demonstrate complementary roles of anterior cingulate and prefrontal cortices in attentional control. NeuroImage. 18(2):483–493. 10.1016/S1053-8119(02)00050-2. 12595201

[bhw350C101] Milham MP, Banich MT 2005 Anterior cingulate cortex: an fMRI analysis of conflict specificity and functional differentiation. Hum Brain Mapp. 25 (3):328–35. 10.1002/hbm.20110. 15834861 PMC6871683

[bhw350C52] Milner B 1963 Effects of different brain lesions on card sorting: the role of the frontal lobes. Arch Neurol. 9:90–100.

[bhw350C53] Monchi O, Petrides M, Petre V, Worsley K, Dagher A 2001 Wisconsin card sorting revisited: distinct neural circuits participating in different stages of the task identified by event-related functional magnetic resonance imaging. J Neurosci. 21(19):7733–7741. 11567063 10.1523/JNEUROSCI.21-19-07733.2001 PMC6762921

[bhw350C54] Nakamura K, Roesch MR, Olson CR 2005 Neuronal activity in macaque SEF and ACC during performance of tasks involving conflict. J Neurophysiol. 93:884–908. 15295008 10.1152/jn.00305.2004

[bhw350C55] Nee DE, Kastner S, Brown JW 2010 Functional heterogeneity of conflict, error, task-switching, and unexpectedness effects within medial prefrontal cortex. NeuroImage. 1–13. 10.1016/j.neuroimage.2010.08.027. PMC2962721 20728547

[bhw350C56] Neta M, Miezin FM, Nelson SM, Dubis JW, Dosenbach NUF, Schlaggar BL, Petersen SE 2015 Spatial and temporal characteristics of error-related activity in the human brain. J Neurosci. 35(1):253–266. 10.1523/JNEUROSCI.1313-14.2015. 25568119 PMC4287146

[bhw350C57] Neta M, Nelson SM, Petersen SE 2016 Dorsal anterior cingulate, medial superior frontal cortex, and anterior insula show performance reporting-related late task control signals. Cereb Cortex. 1–12. 10.1093/cercor/bhw053. 26972752 PMC6059248

[bhw350C58] Neta M, Schlaggar BL, Petersen SE 2014 Separable responses to error, ambiguity, and reaction time in cingulo-opercular task control regions. NeuroImage. 99:59–68. 10.1016/j.neuroimage.2014.05.053. 24887509 PMC4148211

[bhw350C59] Ongür D, Ferry AT, Price JL 2003 Architectonic subdivision of the human orbital and medial prefrontal cortex. J Comp Neurol. 460(3):425–449. 10.1002/cne.10609. 12692859

[bhw350C60] Peterson B, Kane M, Alexander G 2002 An event-related functional MRI study comparing interference effects in the Simon and Stroop tasks. Cogn Brain Res. 13:427–440. 10.1016/s0926-6410(02)00054-x 11919006

[bhw350C61] Ridderinkhof K, Ullsperger M 2004 The role of the medial frontal cortex in cognitive control. Science. 443(2004). 10.1126/science.1100301. 15486290

[bhw350C99] Rordern K, Karnath H-O, Bonilha L 2007 Improving lesion-symptom mapping. J Cogn Neurosci. 19:1081–1088. 17583985 10.1162/jocn.2007.19.7.1081

[bhw350C62] Rudebeck PH, Behrens TE, Kennerley SW, Baxter MG, Buckley MJ, Walton ME, Rushworth MFS 2008 Frontal cortex subregions play distinct roles in choices between actions and stimuli. J Neurosci. 28(51):13775–13785. 10.1523/JNEUROSCI.3541-08.2008. 19091968 PMC6671924

[bhw350C63] Rushworth MFS, Behrens TEJ 2008 Choice, uncertainty and value in prefrontal and cingulate cortex. Nat Neurosci. 11(4):389–397. 10.1038/nn2066. 18368045

[bhw350C64] Schall JD, Emeric EE 2010 Conflict in cingulate cortex function between humans and macaque monkeys: more apparent than real. Brain Behav Evol. 75(4):237–238. 10.1159/000313862. 20693781 PMC3202920

[bhw350C65] Sheth SA, Mian MK, Patel SR, Asaad WF, Williams ZM, Dougherty DD, Bush G, Eskandar EN 2012 Human dorsal anterior cingulate cortex neurons mediate ongoing behavioural adaptation. Nature. 488(7410):218–221. 10.1038/nature11239. 22722841 PMC3416924

[bhw350C66] Silton RL, Heller W, Towers DN, Engels AS, Spielberg JM, Edgar JC, Sass SM, Stewart JL, Sutton BP, Brnich MT, et al 2010 The time course of activity in dorsolateral prefrontal cortex and anterior cingulate cortex during top-down attentional control. NeuroImage. 50(3):1292–1302. 10.1016/j.neuroimage.2009.12.061. 20035885 PMC5998337

[bhw350C67] Silvetti M, Alexander W, Verguts T, Brown JW 2014 From conflict management to reward-based decision making: actors and critics in primate medial frontal cortex. Neurosci Biobehav Rev. 46(P1):44–57. 10.1016/j.neubiorev.2013.11.003. 24239852

[bhw350C68] Simon JR 1990 Effect of conflicting cues on information processing: the “Stroop effect” vs. the ““Simon effect. Acta Psychol (Amst). 73:159–170. 2343770 10.1016/0001-6918(90)90077-s

[bhw350C69] Simon JR, Small AM 1969 Processing auditory information: interference from an irrelevant cue. J Appl Psychol. 53(5):433–435. 5366316 10.1037/h0028034

[bhw350C70] Simon J, Sly P, Vilapakkam S 1981 Effect of compatibility of SR mapping on reactions toward the stimulus source. Acta Psychol (Amst). 47:63–81. 7211438 10.1016/0001-6918(81)90039-1

[bhw350C71] Sohn M-H, Albert MV, Jung K, Carter CS, Anderson JR 2007 Anticipation of conflict monitoring in the anterior cingulate cortex and the prefrontal cortex. Proc Natl Acad Sci U S A. 104(25):10330–10334. 10.1073/pnas.0703225104. 17563353 PMC1965513

[bhw350C72] Specht K, Lie C-H, Shah NJ, Fink GR 2009 Disentangling the prefrontal network for rule selection by means of a non-verbal variant of the Wisconsin Card Sorting Test. Hum Brain Mapp. 30(5):1734–1743. 10.1002/hbm.20637. 18729079 PMC6870978

[bhw350C73] Stürmer B, Redlich M, Irlbacher K, Brandt S 2007 Executive control over response priming and conflict: a transcranial magnetic stimulation study. Exp Brain Res. 183(3):329–339. 10.1007/s00221-007-1053-6. 17643233

[bhw350C74] Stuss DT, Floden D, Alexander MP, Levine B, Katz D 2001 Stroop performance in focal lesion patients: dissociation of processes and frontal lobe lesion location. Neuropsychologia. 39(8):771–786. 11369401 10.1016/s0028-3932(01)00013-6

[bhw350C75] Stuss DT, Kaplan EF, Benson DF, Weir WS, Chiulli S, Sarazin FF 1982 Evidence for the involvement of orbitofrontal cortex in memory functions: an interference effect. J Comp Physiol Psychol. 96(6):913–925. 7153388 10.1037/0735-7036.96.6.913

[bhw350C76] Swick D, Jovanovic J 2002 Anterior cingulate cortex and the Stroop task: neuropsychological evidence for topographic specificity. Neuropsychologia. 40:1240–1253. 11931927 10.1016/s0028-3932(01)00226-3

[bhw350C77] Swick D, Turken AU 2002 Dissociation between conflict detection and error monitoring in the human anterior cingulate cortex. Proc Natl Acad Sci U S A. 99(25):16354–16359. 10.1073/pnas.252521499. 12456882 PMC138615

[bhw350C78] van Veen V, Carter CS 2005 Separating semantic conflict and response conflict in the Stroop task: a functional MRI study. NeuroImage. 27(3):497–504. 10.1016/j.neuroimage.2005.04.042. 15964208

[bhw350C79] Vendrell P, Junqué C, Pujol J, Jurado M 1995 The role of prefrontal regions in the Stroop task. Neuropsychologia. 33(3):341–352. 7792000 10.1016/0028-3932(94)00116-7

[bhw350C97] Vogt B, Nimchinsky EA, Vogt LJ, Hof PR 1995 Human cingulate cortex: surface features, flat maps, and cytoarchitecture. J Comp Neurol. 359 (3):490–506. 10.1002/cne.903590310. 7499543

[bhw350C80] Wang C, Ulbert I, Schomer DL, Marinkovic K, Halgren E 2005 Responses of human anterior cingulate cortex microdomains to error detection, conflict monitoring, stimulus-response mapping, familiarity, and orienting. J Neurosci. 25(3):604–613. 10.1523/JNEUROSCI.4151-04.2005. 15659596 PMC6725336

[bhw350C81] Weissman D, Giesbrecht B, Song A, Mangun G, Woldorff M 2003 Conflict monitoring in the human anterior cingulate cortex during selective attention to global and local object features. NeuroImage. 19(4):1361–1368. 10.1016/S1053-8119(03)00167-8. 12948694

[bhw350C82] Weissman DH, Carp J 2013 The Congruency effect in the posterior medial frontal cortex is more consistent with time on task than with response conflict. PLoS ONE. 8(4). 10.1371/journal.pone.0062405. PMC3639275 23638070

[bhw350C83] West R, Travers S 2008 Tracking the temporal dynamics of updating cognitive control: an examination of error processing. Cereb Cortex. 18(5):1112–1124. 10.1093/cercor/bhm142. 17716989

[bhw350C84] Wilk HA, Ezekiel F, Morton JB 2012 Brain regions associated with moment-to-moment adjustments in control and stable task-set maintenance. NeuroImage. 59(2):1960–1967. 10.1016/j.neuroimage.2011.09.011. 21945693

[bhw350C100] Wühr P, Ansorge U 2005 Exploring trial-by-trial modulation of the Simon effect. Psychon Bull Rev. 12 (2):282–8. 16082807 10.3758/bf03196373

[bhw350C85] Yeung N, Cohen JD, Botvinick MM 2011 Errors of interpretation and modeling: a reply to Grinband et al. NeuroImage. 57(2):316–319. 10.1016/j.neuroimage.2011.04.029. 21530662 PMC3737739

