# Supplementary material for: Distinct Roles for the Anterior Cingulate and Dorsolateral Prefrontal Cortices During Conflict Between Abstract Rules
Source: Cereb Cortex. 2016 Nov 22;27(1):34–45. doi: 10.1093/cercor/bhw350 (PMC5939207; doi:10.1093/cercor/bhw350)
Supplement: Supplementary Data [file boschinetalcc2016_supplementarymaterial.pdf]

Supplementary Material

REGIONS OF INTEREST (ROI)

DLPFC | ACC

Brodmann areas

ID	8L	8R	9L	9R	46L	46R	TOTAL	24L	24R	32L	32R	TOTAL	TOTAL ROI	TOTAL VOL
1	0	6072	0	2880	0	104	9056	0	1272	0	5048	6320	15376	30192
2	96	0	5416	0	1520	0	7032	0	0	4128	0	4128	11160	20864
3	0	0	0	0	0	0	0	2285	1540	1489	2001	7315	7315	39014
4	0	136	0	2432	0	5320	7888	0	0	0	0	0	7888	49984
5	0	0	32	0	464	0	496	0	0	0	0	0	496	1384
6	0	0	3144	0	6760	0	9904	0	0	1040	0	1040	10944	34960

Lesion volumes in cubic mm

Overall regions of interest

ID	OUT of ROIs VOLUME		OUT OF TOTAL VOLUME	
	DLPFC	ACC	DLPFC	ACC
1	59%	41%	30%	21%
2	63%	37%	34%	20%
3	0%	100%	0%	19%
4	100%	0%	16%	0%
5	100%	0%	36%	0%
6	90%	10%	28%	3%

Specific Brodmann areas of interest

ID	OUT OF ROIs VOLUME		OUT OF TOTAL VOLUME	
	46	24+32	46	24+32
1	1%	41%	0%	21%
2	14%	37%	7%	20%
3	0%	100%	0%	19%
4	67%	0%	11%	0%
5	94%	0%	34%	0%
6	62%	10%	19%	3%

Table S1. For each patient (ACC group = green, dlPFC group = blue), lesion volumes in mm3 are reported for each general region of interest (ACC and dlPFC) and for specific Brodmann areas within the ROIs. Total ROI volume in the second rightmost column is calculated as the sum of the volume of damage across all general ROIs. Total volume in the rightmost column refers to the total volume of damage across the whole brain.

Overall regions of interest

ID	OUT of ROIs VOLUME		OUT OF TOTAL VOLUME	
	DLPFC	ACC	DLPFC	ACC
1	59%	41%	30%	21%
2	63%	37%	34%	20%
3	0%	100%	0%	19%
4	100%	0%	16%	0%
5	100%	0%	36%	0%
6	90%	10%	28%	3%

Table S2. Percentage of lesion localized to each specific ROIs out of the lesion volume across all general ROIs (left) and out of total brain damage (right) for each patient.

Specific Brodmann areas of interest

ID	OUT OF ROIs VOLUME		OUT OF TOTAL VOLUME	
	46	24+32	46	24+32
1	1%	41%	0%	21%
2	14%	37%	7%	20%
3	0%	100%	0%	19%
4	67%	0%	11%	0%
5	94%	0%	34%	0%
6	62%	10%	19%	3%

Table S3. Percentage of lesion localized to each specific Brodmann area of interest out of the lesion volume across all general ROIs (left) and out of total brain damage (right) for each patient.
